# A nomogram for predicting ischaemic muscle sequelae after revascularization in patients with traumatic femoral-popliteal artery injuries: a retrospective cohort study

**DOI:** 10.1007/s00264-025-06470-y

**Published:** 2025-04-07

**Authors:** Huiyang Jia, Heng Zhang, Lin Jin, Haofei Wang, Qi Dong, Wei Chen, Yingze Zhang, Lin Liu, Zhiyong Hou

**Affiliations:** 1 Department of Orthopaedic Surgery, Hebei Medical University Third Hospital, Shijiazhuang, China; 2 Engineering Research Center of Orthopedic MinimallyInvasive Intelligent Equipment, Ministry of Education, Shijiazhuang, China; 3 Key Laboratory of Biomechanics of Hebei Province, Shijiazhuang, China; 4 NHC Key Laboratory of Intelligent Orthopaedic Equipment, Shijiazhuang, China; 5 Key Laboratory of Precise Assessment, Diagnosis, and Treament of Soft Tissue Injury of Hebei Province, Shijiazhuang, China

**Keywords:** Artery injury, Ischaemic muscle sequelae, Predictors, Nomogram

## Abstract

**Purpose:**

This study aimed to investigate the incidence and associated risk factors of ischaemic muscle sequelae in patients with traumatic femoropopliteal artery injuries following revascularization, as well as to develop a nomogram to predict the risk of ischaemic muscle sequelae.

**Methods:**

Data from patients with acute traumatic femoropopliteal artery injuries between January 2008 and December 2022 were collected. All patients with successful limb salvage were divided into two groups based on the occurrence of ischaemic muscle sequelae: the ischemic muscle sequelae group (IG) and the non-ischaemic muscle sequelae group (NG). Univariate and multivariate logistic regression analyses were used to identify potential predictive factors associated with ischaemic muscle sequelae. A predictive nomogram was constructed and internally validated.

**Results:**

Among the 102 patients, 30 cases (29.41%) developed ischaemic muscle sequelae. Independent predictors of ischaemic muscle sequelae were identified as crush injury, HCT, and CKMB. A nomogram was constructed based on these three parameters. The area under the receiver operating characteristic (ROC) curve of the predictive model was 0.894, indicating excellent discrimination. The calibration curve demonstrated a high degree of consistency between the predicted probabilities and the observed outcomes. Additionally, the decision curve analysis (DCA) showed that the nomogram model had good predictive capability.

**Conclusions:**

Our study demonstrated that crush injury, HCT, and CKMB were independent predictors of ischaemic muscle sequelae in patients with acute traumatic femoropopliteal artery injuries following revascularization. The nomogram integrating clinical factors and blood markers can assist physicians in conveniently predicting the risk of ischaemic muscle sequelae in patients.

## Introduction

Trauma is a major global public health issue, with vascular injuries accounting for 0.65–1.14% [1, 2]. Lower extremity arterial injuries make up approximately 30–40% of all peripheral arterial injuries [3–5]. Although the incidence of vascular injuries is relatively low, they can lead to serious complications and remain a challenge for professionals at trauma centres. If not promptly detected and treated, major arterial injuries in the lower limbs can lead to ischaemia, limb loss, and even life-threatening conditions. In cases of severe injury or prolonged ischaemia, the trauma team often performs primary amputation to save the patient’s life or avoid futile treatment [6]. However, patients who do not require immediate primary amputation may later need delayed amputation due to irreversible or progressive ischaemia, uncontrolled infection, or irreparable soft tissue or skeletal damage [3]. Even patients who successfully retain their limbs may experience functional impairments, such as ischaemic muscle sequelae in the muscles [7].

The current literature on vascular injury prognosis primarily focuses on severe complications such as death and amputation [8–10]. With advancements in trauma care systems, surgical techniques, and the rapid development of endovascular devices, patient mortality and amputation rates have significantly decreased [11–13]. Clinicians are increasingly focusing on the functional prognosis of patients [14, 15]. Ischaemia-induced muscle tissue necrosis, followed by ischaemic sequelae such as fibrosis and contracture, significantly impairs the functional prognosis of patients.

A nomogram is a graphical tool that combines multiple risk factors in an intuitive way to predict the likelihood of an event occurring, and it is widely used in predictive medicine [16, 17]. However, no studies have yet used a nomogram to predict the risk of ischaemic muscle sequelae in patients with traumatic femoropopliteal arterial injuries. Therefore, our study aims to investigate the incidence of ischaemic muscle sequelae and their associated risk factors, as well as to develop a nomogram to predict the risk of ischaemic muscle sequelae in patients with traumatic femoropopliteal arterial injuries.

## Patients and methods

### Patients

This retrospective study was approved by the institutional review board of our hospital (Ke 2023-107-1) before data collection. The study subjects were patients with acute traumatic femoropopliteal artery injuries who received treatment at our hospital from January 2008 to December 2022. Inclusion criteria were: (1) acute traumatic femoral artery injury; (2) acute traumatic popliteal artery injury. Exclusion criteria were: (1) patients who did not undergo emergency arterial exploration and repair; (2) patients who died during hospitalization; (3) patients who underwent primary amputation or delayed amputation; (4) Patients with nerve injuries; (5) incomplete medical records; (6) lost to follow-up. Patients with ischaemic muscle necrosis exhibited muscle atrophy, increased stiffness, and restricted joint mobility during the follow-up period. All patients were followed up for at least one year after discharge and were divided into groups with ischaemic muscle sequelae (IG) and without ischaemic muscle sequelae (NG).

Demographic information, comorbidities, trauma presentation, admission laboratory test results, and surgical interventions were recorded for clinical and statistical assessment. Demographic data included age, gender, body mass index (BMI, kg/m2), smoking (yes vs. no), alcohol (yes vs. no). Comorbidities included a history of arrhythmia, coronary artery disease, hypertension, diabetes, and cerebral infarction. Trauma presentation included side of injury, the mechanism of injury (car crash injury, high fall injury, crush injury, same-level fall, sharp injury, unknown trauma), injured blood vessel (femoral artery, popliteal artery), status of the blood vessel injury site (closed fracture or dislocation, open fracture or dislocation, simple open wound, simple soft tissue injury), tibiofibular fracture (closed, open, no), lower leg degloving or open soft tissue injury (yes vs. no), collateral circulation (yes vs. no), Mangled Extremity Severity Score (MESS), types of arterial injury (thrombosis, partial arterial rupture, complete arterial rupture) and venous injury (yes vs. no). Surgical intervention data included ischaemia time (hours), surgical approach (end-to-end anastomosis, great saphenous vein grafting, artificial vascular grafting, interventional covered stent placement, thrombus aspiration, vascular compression release), emergency surgery time(hours), fasciotomy (yes vs. no), vascular reoperation (yes vs. no) and secondary infections (yes vs. no). Admission laboratory parameters included white blood cells (WBC, 10^9^/L), neutrophils (NEU, 10^9^/L), lymphocytes (LYM, 10^9^/L), monocytes (MON, 10^9^/L), eosinophils (EOS, 10^9^/L), basophils (BAS, 10^9^/L), red blood cells (RBC, 10^12^/L), haemoglobin (HGB, g/L), haematocrit (HCT, %), platelets (PLT, 10^9^/L), international normalized ratio (INR, s), prothrombin time (PT, s), activated partial thromboplastin time (APTT, s), fibrinogen (FIB, g/L), thrombin time (TT, s), D-dimer (mg/L), total protein (TP, g/L), albumin (ALB, g/L), globulin (GLOB, g/L), alanine aminotransferase (ALT, U/L), aspartate aminotransferase (AST, U/L), alkaline phosphatase (ALP, U/L), cholinesterase (CHE, KU/L), creatine kinase (CK, U/L), creatine kinase-MB (CKMB, U/L), lactate dehydrogenase (LDH, U/L), triglycerides (TG, mmol/L), sodium (Na⁺, mmol/L), potassium (K⁺, mmol/L), calcium (Ca²⁺, mmol/L), chloride (Cl⁻, mmol/L), total carbon dioxide (TCO₂, mmol/L), glucose (GLU, mmol/L), urea (UREA, mmol/L), creatinine (CREA, µmol/L), osmolality (OSM, mOsm/L) and uric acid (UA, µmol/L).

### Statistical analysis

Continuous variables were presented as mean ± standard deviation or median and interquartile range (IQR), depending on data distribution, and were assessed using the Student’s t-test or Mann-Whitney U test as appropriate. Categorical variables were presented as numbers and were evaluated using the chi-square test or Fisher’s exact test, as appropriate. Univariate logistic regression analysis was conducted to identify factors associated with ischemic muscle sequelae. Variables with statistical significance in univariate analysis were included in multivariate logistic regression analysis. A nomogram was developed based on the results of the multivariate logistic regression analysis. Internal validation of the nomogram was performed using the bootstrap method with 1000 resamples to assess discrimination and calibration [18]. The area under the receiver operating characteristic (ROC) curve (AUC) was used to evaluate the predictive accuracy of the model. A calibration plot was used to graphically assess the relationship between observed and predicted probabilities of ischemic muscle sequelae. To evaluate the clinical utility of the nomogram, we used decision curve analysis (DCA) to estimate the net benefit and threshold probability. All statistical tests were two-sided, and a P-value of less than 0.05 was considered statistically significant. Analyses were conducted using IBM SPSS Statistics (version 25.1, IBM Corp, Armonk, NY, USA) and the “rms” package in R software (version 4.2.0, R Foundation for Statistical Computing, Vienna, Austria, http://www.R-project.org) for constructing the nomogram.

## Results

### Baseline characteristics

**Fig. 1:**
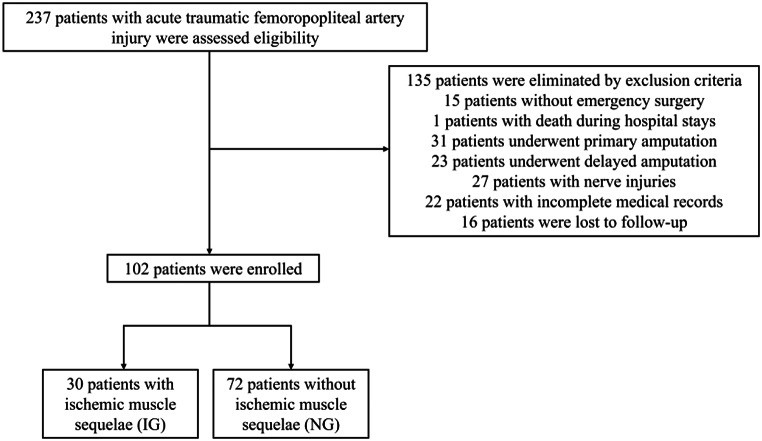
Flow diagram of included patients

From January 2008 to December 2022, a total of 237 patients with acute traumatic femoropopliteal artery injuries were admitted. 15 patients who did not undergo emergency surgical exploration and arterial repair, one patient who died during hospitalization, 31 patients who underwent primary amputation due to severe limb destruction, 23 patients who had delayed amputation, 27 patients with nerve injuries, 22 patients with incomplete medical records, and 16 patients lost to follow-up were excluded. Finally, 102 eligible patients with femoropopliteal artery injuries were included (Fig. 1). Among the 102 patients, 30 had ischaemic muscle sequelae, while 72 did not, resulting in an incidence of ischaemic muscle sequelae among patients with femoropopliteal artery injuries of 29.41%. A total of 37 patients with femoral artery injuries and 65 patients with popliteal artery injuries were included, with no statistically significant difference between the two groups. Of the 102 patients, 83 were male (81.37%) and 19 were female (18.63%). The overall mean age was 42.98 ± 17.21 years. Except for a history of cerebral infarction (*P* = 0.024), there were no statistically significant differences in demographic information or comorbidities between the two groups (Table 1).

**Table 1:** Clinical characteristics of patients with acute traumatic femoral-popliteal artery injuries in the two groups

Variable	Non-Ischemic muscle sequelae group (*n* = 30)	Ischemic muscle sequelae group (*n* = 72)	*P* Value
Age, years	45.0 (27)	46.0 (28)	0.423
Gender			0.577
Male	57	26	
Female	15	4	
BMI (kg/m^2^)	25.39 ± 4.11	26.23 ± 3.67	0.314
Smoking			0.618
Yes	16	8	
No	56	22	
Alcohol			0.442
Yes	18	5	
No	54	25	
Arrhythmia			1
Yes	1	0	
No	71	30	
Coronary heart disease			1
Yes	1	0	
No	71	30	
Hypertension			0.763
Yes	10	5	
No	62	25	
Diabetes			1
Yes	3	1	
No	69	29	
Cerebral Infarction			0.024
Yes	0	3	
No	72	27	
Side of injury			1
Left	35	14	
Right	37	16	
Mechanism of injury			< 0.0001
Car crash injury	26	7	
High fall injury	2	0	
Crush injury	9	18	
Same-level fall	9	2	
Sharp injury	17	3	
Unknown trauma	4	0	
Crush injury			< 0.0001
Yes	9	18	
No	63	12	
Injured blood vessel			1
Femoral artery	26	11	
Popliteal artery	46	19	
Status of the blood vessel injury site			0.530
Closed fracture or dislocation	30	15	
Open fracture or dislocation	7	4	
Simple open wound	25	6	
Simple soft tissue injury	10	5	
Tibiofibular fracture			0.202
Closed	2	3	
Open	4	3	
No	66	24	
lower leg degloving or open soft tissue injury			0.329
Yes	7	5	
No	65	25	
Collateral circulation			1
Yes	18	7	
No	54	23	
MESS	5.0 (1)	6.0 (2)	0.046
Types of arterial injury			0.568
Thrombosis	35	15	
Partial arterial rupture	10	2	
Complete arterial rupture	27	13	
Venous injury			0.819
Yes	24	9	
No	48	21	
Ischaemia time (hours)	10.0 (8.75)	12.0 (11.25)	0.079
Surgical approach			0.357
End-to-end anastomosis	33	11	
Great saphenous vein grafting	22	7	
Artificial vascular grafting	5	4	
Interventional covered stent placement	7	7	
Thrombus aspiration	3	1	
Vascular compression release	2	0	
Emergency surgery time(hours)	3.87 (2.38)	3.0 (2.07)	0.433
Fasciotomy			0.038
Yes	18	14	
No	54	16	
Vascular reoperation			0.744
Yes	8	4	
No	64	26	
Secondary infection			0.002
Yes	13	15	
No	59	15	
WBC,10^9^/L	11.75 (7.50)	11.36 (7.68)	0.641
NEU,10^9^/L	9.82 (8.25)	9.82 (7.31)	0.430
LYM,10^9^/L	1.35 (1.30)	1.08 (0.71)	0.014
MON,10^9^/L	0.74 (0.72)	0.75 (0.45)	0.569
EOS,10^9^/L	0.08 (0.14)	0.01 (0.11)	0.005
BAS,10^9^/L	0.02 (0.05)	0.01 (0.01)	0.045
RBC,10^12^/L	3.76 ± 0.73	3.33 ± 0.99	0.037
HGB, g/L	115.39 ± 20.15	102.41 ± 24.35	0.013
HCT, %	34.46 ± 6.05	29.65 ± 7.25	0.002
PLT,10^9^/L	209.15 ± 83.76	170.70 ± 68.17	0.018
INR, s	1.14 (0.13)	1.13 (0.22)	0.903
PT, s	13.25 (2.07)	13.55 (1.63)	0.351
APTT, s	28.70 (5.77)	29.9 (7.3)	0.497
FIB, g/L	2.48 (1.2)	2.52 (1.79)	0.906
TT, s	15.65 (3.27)	15.55 (3.43)	0.834
D-dimer, mg/L	1.74 (7.75)	2.66 (8.54)	0.569
TP, g/L	59.3 (12.28)	52.10 (19.75)	0.061
ALB, g/L	36.0 (8.99)	33.44 (15.23)	0.467
GLOB, g/L	23.08 (4.22)	21.15 (5.46)	0.013
ALT, U/L	28.0 (23.8)	30.0 (40.5)	0.544
AST, U/L	32.0 (25)	57.5 (178.8)	0.004
ALP, U/L	57.5 (29)	55.5 (31)	0.189
CHE, KU/L	6.44 ± 1.86	5.75 ± 1.90	0.099
CK, U/L	547.5 (1402.6)	1760.85(7315.3)	0.0004
CKMB, U/L	23.5 (28.8)	78.065(441.69)	< 0.0001
LDH, U/L	347.0 (289.39)	413.5 (419.25)	0.282
TG, mmol/L	0.85 (0.85)	0.81 (0.75)	0.210
Na^+^,mmol/L	139.24 (4.68)	137.1 (3.08)	0.099
K^+^,mmol/L	3.89 ± 0.48	3.86 ± 0.38	0.758
Ca^2+^,mmol/L	2.12 (0.24)	2.13 (0.31)	0.336
Cl^−^,mmol/L	105.35 (4.55)	105.45 (6.65)	0.646
TCO2,mmol/L	23.015 (4.4)	23.0(4.18)	0.513
GLU, mmol/L	7.11 (2.58)	7.41 (3)	0.481
UREA, mmol/L	5.06 ± 1.52	5.70 ± 1.96	0.120
CREA,µmol/L	63.84 ± 15.20	62.04 ± 13.04	0.549
OSM, mOsm/L	273.5 (25.63)	287.9 (26.67)	0.049
UA,µmol/L	318.10 ± 111.79	270.57 ± 84.37	0.022

### The univariate and multivariate logistic regression analysis of risk factors for ischaemic muscle sequelae

Univariate logistic regression analysis identified 14 factors significantly associated with ischaemic muscle sequelae, including risk factors such as crush injury, secondary infection, CK, CKMB, AST, and fasciotomy, and protective factors such as HCT, HGB, GLOB, LYM, RBC, EOS, PLT, and UA (Table 2). These candidate predictors were then included in a multivariate logistic regression analysis. The results of the multivariate logistic regression analysis are shown in Table 3. Multivariate analysis revealed that crush injury (odds ratio [OR] = 18.862, 95% confidence interval [CI] = 2.970–119.797, *P* = 0.002) and CKMB (OR = 1.057, 95% CI = 1.008–1.109, *P* = 0.022) were independent risk factors for ischaemic muscle sequelae. HCT (OR = 0.645, 95% CI = 0.457–0.912, *P* = 0.013) was identified as an independent protective factor for ischaemic muscle sequelae.

**Table 2:** Univariate logistic regression analysis of risk factors for ischemic muscle sequelae

Variable	OR	95%CI		PValue
		Lower limit	Upper limit	
Crush injury	10.5	3.945	30.189	< 0.0001
Secondary infection	4.538	1.801	11.802	0.002
CK, U/L	1.001	1.000	1.001	0.002
HCT, %	0.894	0.828	0.957	0.002
CKMB, U/L	1.022	1.010	1.039	0.003
HGB, g/L	0.973	0.952	0.993	0.010
GLOB, g/L	0.877	0.787	0.966	0.011
AST, U/L	1.006	1.002	1.011	0.014
LYM,10^9^/L	0.435	0.199	0.789	0.019
RBC,10^12^/L	0.499	0.267	0.873	0.020
EOS,10^9^/L	0.001	0.000	0.264	0.031
PLT,10^9^/L	0.994	0.987	0.999	0.033
Fasciotomy	2.625	1.073	6.480	0.034
UA,µmol/L	0.995	0.990	0.999	0.043

**Table 3:** Multivariate logistic regression analysis of risk factors for ischemic muscle sequelae

Variable	OR	95%CI		PValue
		Lower limit	Upper limit	
Crush injury	18.862	2.970	119.797	0.002
HCT, %	0.645	0.457	0.912	0.013
CKMB, U/L	1.057	1.008	1.109	0.022

### Development and validation of a nomogram for predicting ischaemic muscle sequelae

**Fig. 2:**
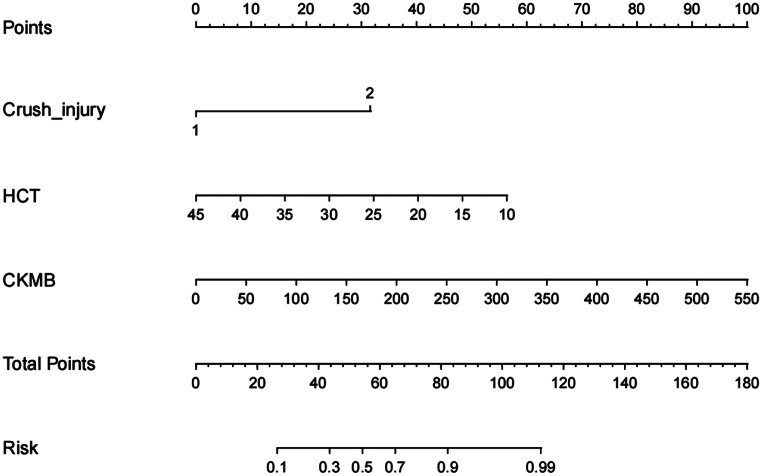
A nomogram for predicting ischemic muscle sequelae following revascularization in patients with acute traumatic femoropopliteal artery injuries

**Fig. 3:**
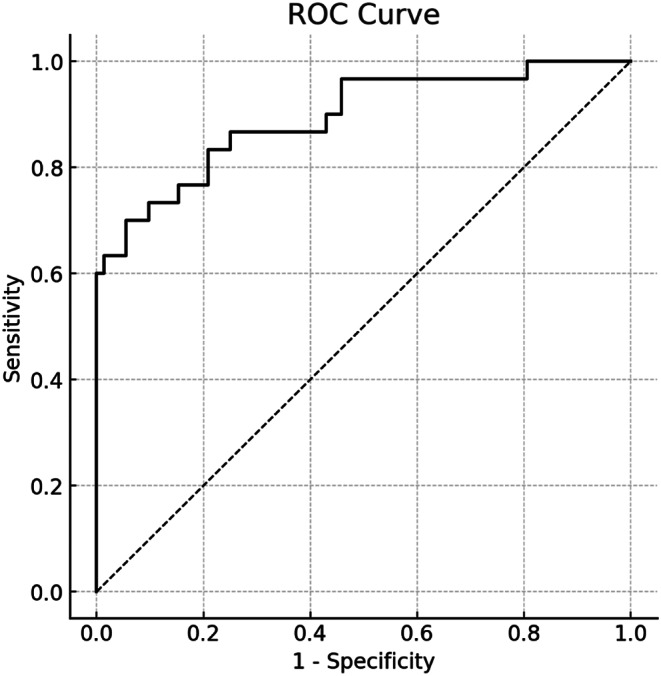
Receiver operating characteristic (ROC) curves for the predictive model

**Fig. 4:**
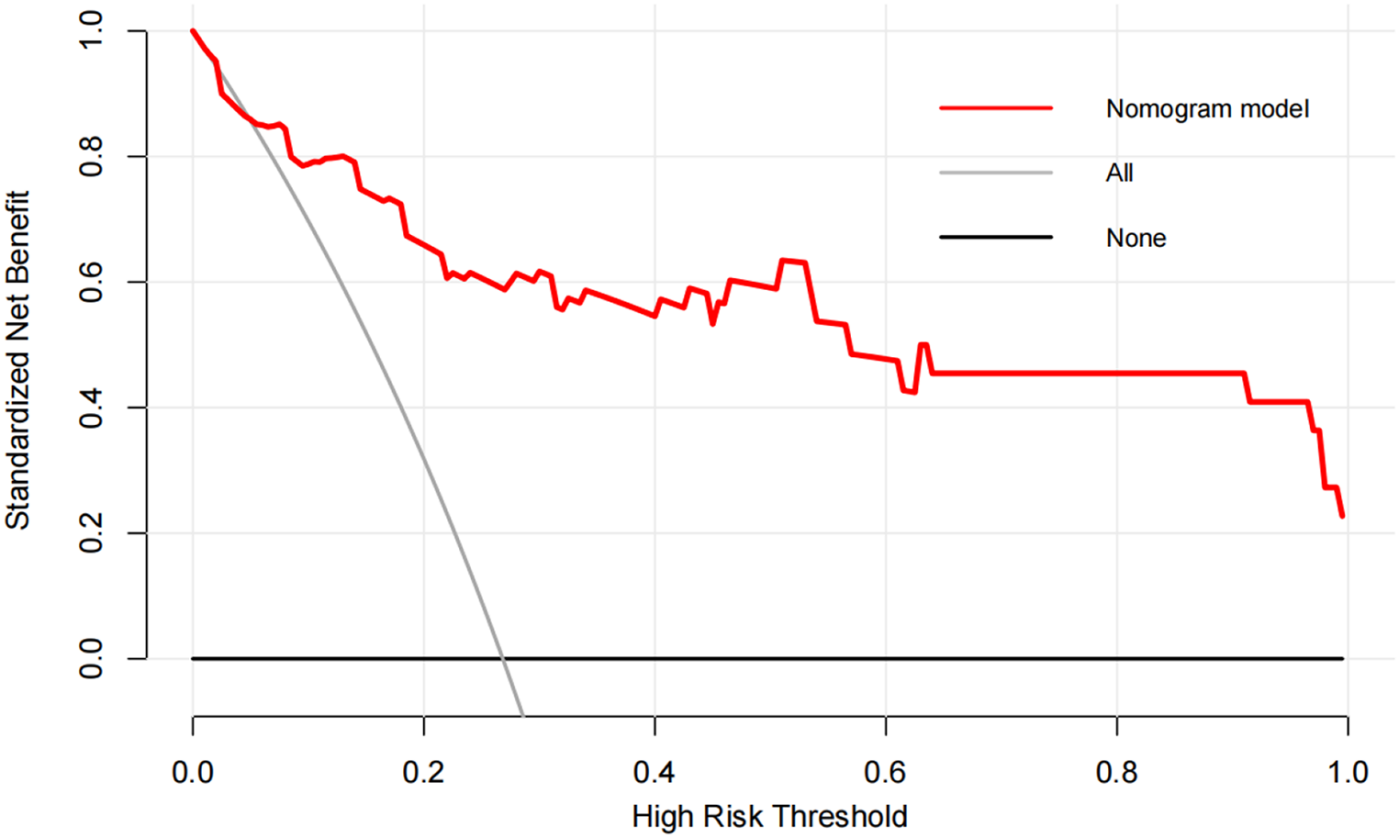
Calibration plot in the internal validation

**Fig. 5:**
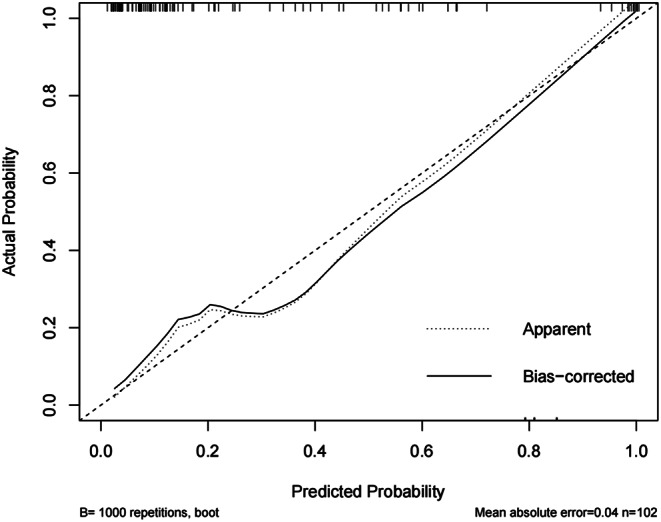
Decision curve analysis for nomogram prediction model

A nomogram was developed to predict ischaemic muscle sequelae following revascularization in traumatic femoropopliteal artery injury, based on three independent predictive factors identified by multivariate logistic regression analysis: crush injury, HCT, and CKMB (Fig. 2). The model including these three variables showed good overall predictive accuracy (AUC = 0.894, 95% CI = 0.819–0.968) (Fig. 3). Furthermore, the calibration plot in the internal validation indicated good correlation between the observed probability and the predicted probability of ischaemic muscle sequelae, with an adjusted C-statistic of 0.879 (Fig. 4). The DCA curve showed that the nomogram model had good predictive capability (Fig. 5).

## Discussion

Arterial injuries in limbs often present as complex injury patterns, posing unique challenges for management and potentially leading to severe outcomes such as death or amputation [19, 20]. Survivors typically experience poor functional outcomes and face long-term disabilities. The current literature primarily focused on risk factors for amputation in femoral-popliteal artery injuries, with fewer studies examining risk factors for functional outcomes like ischaemic muscle sequelae [5, 21]. The functional outcomes of vascular injury patients are not only closely related to timely surgical intervention to restore blood supply but also strongly associated with the severity of the initial injury. Admission laboratory tests can effectively reflect the extent of trauma in patients; thus, the results of these tests may also serve as predictors of patient prognosis to a certain extent. A review of relevant studies revealed that previous research had primarily focused on trauma-related factors, while the predictive value of laboratory indicators at admission had been largely overlooked. Therefore, our study aimed to investigate the risk factors for ischaemic muscle dysfunction following revascularization in patients with traumatic femoropopliteal artery injuries using comprehensive data, including demographics, comorbidities, trauma characteristics, admission laboratory results, and surgical interventions. In addition, we sought to develop corresponding nomograms for clinical application to assist clinicians in identifying high-risk patients and providing timely management.

In this study, we investigated the associations between ischaemic muscle sequelae following revascularization of femoropopliteal artery injuries and demographic information, comorbidities, trauma characteristics, admission laboratory results, and surgical interventions. Crush injury, HCT, and CKMB were identified as independent predictive factors for ischaemic muscle sequelae. Furthermore, we developed a nomogram to predict the occurrence of ischaemic muscle sequelae following revascularization in patients with femoropopliteal artery injuries based on these three identified risk factors. The nomogram demonstrated good accuracy in assessing the risk of ischaemic muscle sequelae. In addition, the nomogram was internally validated and showed good performance in both calibration and discrimination.

Crush injury is widely recognized as a severe type of trauma that is prone to muscle necrosis. Crush injuries and vascular injuries share a similar pathophysiological mechanism, commonly referred to as ischaemia-reperfusion injury [22]. During reperfusion, the reoxygenation of ischaemic tissues leads to a substantial accumulation of inflammatory cells and the release of large amounts of reactive oxygen species (ROS), hydroxyl radicals, and cytokines, resulting in tissue damage [23]. Furthermore, the permeability of capillary endothelial cells is impaired, contributing to increased tissue oedema and exacerbation of hypoxia. Additionally, crush injuries directly compromise the integrity of the sarcolemma, increasing membrane permeability. The influx of Ca2 + activates Ca2+-dependent phospholipases, leading to the degradation of membrane phospholipids and further damaging the cell membrane and mitochondria. Mitochondrial dysfunction reduces ATP production, while free fatty acids generated during this process contribute to the synthesis of ROS, thereby amplifying the extent of tissue damage [24]. These mechanisms collectively explained the poor prognosis associated with vascular injuries caused by crush injuries. Multiple studies have also demonstrated that the injury mechanisms of blunt trauma, including crush injuries, result in a higher rate of amputations and worse functional outcomes [9, 10, 15, 21]. Therefore, in patients with vascular injuries caused by crush injuries, even after limb salvage, special attention should be paid to the adverse functional outcomes associated with muscle fibrosis and contractures.

Over the years, creatine kinase-MB (CKMB) has been an important diagnostic marker for assessing potential acute myocardial infarction [25]. During skeletal muscle injury, CK expression reverts to an embryonic isoform pattern, with the B chain of CK being re-expressed as part of the repair process [26]. As a result, CKMB has also been used to monitor skeletal muscle injury [27]. Wang et al. reported that CKMB is an independent risk factor for muscle necrosis in patients with acute compartment syndrome (ACS) [28]. However, to the best of our knowledge, no studies have explored the predictive value of CKMB levels for muscle necrosis in patients with arterial injuries. In this study, we investigated the role of initial CKMB levels in predicting ischaemic muscle contractures after revascularization of femoropopliteal artery injuries. Based on multivariable logistic regression analysis, we identified CKMB as an independent risk factor for poor prognosis.

Hematocrit (HCT) is a whole blood parameter that reflects the proportion of red blood cells to plasma. In our study, low HCT values were identified as an independent risk factor for ischemic muscle sequelae. The loss of red blood cells caused by massive hemorrhage, along with the inflammatory response and oxidative stress damage to red blood cells during ischemia-reperfusion injury, may contribute to the outcome of low HCT levels [29]. Therefore, the increased likelihood of ischaemic muscle sequelae in arterial injury patients with low HCT is likely due to insufficient oxygen-carrying capacity and more severe ischemia-reperfusion injury. Similarly, a study by Lin et al. found that patients with acute coronary syndrome who had lower haemoglobin levels were more likely to experience poor outcomes, which may follow the same reasoning [30]. Thus, appropriate blood transfusion in vascular injury patients may improve adverse outcomes of ischaemic muscle sequelae.

Notably, in univariate logistic regression analysis, fasciotomy was significantly associated with ischaemic muscle sequelae (*P* = 0.034), suggesting that fasciotomy may increase the likelihood of ischaemic contracture following revascularization in patients with femoropopliteal artery injuries. Although it did not show significance in the multivariate logistic regression analysis, fasciotomy remains a topic worth exploring. Acute compartment syndrome refers to circulatory impairment within a closed space due to excessively high internal pressure, and ischemia-reperfusion following vascular trauma is an important mechanism in its development [31]. Given the high uncertainty in diagnosing compartment syndrome and the high rates of amputation and mortality associated with misdiagnosis, preventive fasciotomy is often performed in clinical practice to minimize the risk of compartment syndrome secondary to major arterial injuries [32]. In recent years, some researchers have found that the time from injury to fasciotomy does not significantly impact outcomes. Zhang et al. reported that the timing of fasciotomy was not associated with mortality or amputation [33]. Similarly, Mortensen et al. found no difference in the time from injury to fasciotomy between patients with muscle necrosis and those without muscle necrosis [34]. Kauvar et al. reported similar findings in their study on lower extremity vascular injuries in military personnel. They found that early fasciotomy was not associated with amputation but was related to limb infections, functional impairment, and contractures [35]. However, the more severe injury patterns observed in patients undergoing fasciotomy may be an important contributing factor. Therefore, the value of fasciotomy in the treatment of patients with arterial injuries requires further investigation.

Another noteworthy factor that may significantly impact prognosis is ischaemic time. In our analysis, the median ischemic time in the ischaemic muscle sequelae group was 12 (11.25) hours, while it was ten (8.75) hours in the non- ischaemic muscle sequelae group, slightly shorter than that of the ischaemic muscle sequelae group. However, the difference was not statistically significant (*P* = 0.079). This outcome might have been attributed to the small sample size, leading to limited statistical power. Alternatively, it might have reflected that ischaemic time had a significant impact on the likelihood of limb salvage, while the initial injury mechanism exerted a more pronounced influence on the prognosis of muscular function following limb salvage.

This study has several limitations. First, it is a retrospective study, which inherently presents limitations in data collection. Second, the analysis was based on data from a single institution, with a relatively small study population. Therefore, the generalizability of the findings may be limited, necessitating validation through large-scale, multicenter studies. Third, although the accuracy of our nomogram has been extensively validated using bootstrap testing for internal validation, external validation is lacking. Lastly, our retrospective analysis spans a long period, during which advancements in surgical techniques may have influenced the prognosis of patients with femoropopliteal artery injuries.

## Conclusions

Our study demonstrated that crush injury, HCT, and CKMB were independent predictors of ischaemic muscle sequelae in patients with acute traumatic femoropopliteal artery injuries following revascularization. Additionally, we developed a novel nomogram that exhibited excellent predictive capability, enabling clinicians to accurately and timely predict the risk of ischaemic muscle sequelae in patients. Assessing the individual’s risk enables clinicians to more comprehensively implement necessary measures to reduce the incidence of ischaemic muscle sequelae by monitoring the condition and intervening medically.

## Data Availability

No datasets were generated or analysed during the current study.

## Abbreviations

BMI: Body Mass Index
WBC: White Blood Cells
NEU: Neutrophils
LYM: Lymphocytes
MON: Monocytes
EOS: Eosinophils
BAS: Basophils
RBC: Red Blood Cells
HGB: Haemoglobin
HCT: Haematocrit
PLT: Platelets
INR: International Normalized Ratio
PT: Prothrombin Time
APTT: Activated Partial Thromboplastin Time
FIB: Fibrinogen
TT: Thrombin Time
TP: Total Protein
ALB: Albumin
GLOB: Globulin
ALT: Alanine Aminotransferase
AST: Aspartate Aminotransferase
ALP: Alkaline Phosphatase
CHE: Cholinesterase
CK: Creatine Kinase
CKMB: Creatine Kinase-MB
LDH: Lactate Dehydrogenase
TG: Triglycerides
TCO₂: Total Carbon Dioxide
GLU: Glucose
UREA: Urea
CREA: Creatinine
OSM: Osmolality
UA: Uric Acid
IQR: Interquartile Range
ROC: Receiver Operating Characteristic
AUC: Area Under The Curve
DCA: Decision Curve Analysis
ROS: Reactive Oxygen Species
ACS: Acute Compartment Syndrome
